# Development and Validation of a Personality Assessment Instrument for Traditional Korean Medicine: Sasang Personality Questionnaire

**DOI:** 10.1155/2012/657013

**Published:** 2012-04-11

**Authors:** Han Chae, Siwoo Lee, Soo Hyun Park, Eunsu Jang, Soo Jin Lee

**Affiliations:** ^1^Division of Longevity and Biofunctional Medicine, School of Korean Medicine, Pusan National University, Busan 626-870, Republic of Korea; ^2^Korea Institute of Oriental Medicine, Daejeon 305-811, Republic of Korea; ^3^Department of Occupational Therapy, Yonsei University, Wonju 220-710, Republic of Korea; ^4^Department of Psychotherapy, Kyungil University, Daegu 712-701, Republic of Korea

## Abstract

*Objective*. Sasang typology is a traditional Korean medicine based on the biopsychosocial perspectives of Neo-Confucianism and utilizes medical herbs and acupuncture for type-specific treatment. This study was designed to develop and validate the Sasang Personality Questionnaire (SPQ) for future use in the assessment of personality based on Sasang typology. *Design and Methods*. We selected questionnaire items using internal consistency analysis and examined construct validity with explorative factor analysis using 245 healthy participants. Test-retest reliability as well as convergent validity were examined. *Results*. The 14-item SPQ showed acceptable internal consistency (Cronbach's alpha = .817) and test-retest reliability (*r* = .837). Three extracted subscales, SPQ-behavior, SPQ-emotionality, and SPQ-cognition, were found, explaining 55.77% of the total variance. The SPQ significantly correlated with Temperament and Character Inventory novelty seeking (*r* = .462), harm avoidance (*r* = −.390), and NEO Personality Inventory extraversion (*r* = .629). The SPQ score of the So-Eum (24.43 ± 4.93), Tae-Eum (27.33 ± 5.88), and So-Yang (30.90 ± 5.23) types were significantly different from each other (*P* < .01). *Conclusion*. Current results demonstrated the reliability and validity of the SPQ and its subscales that can be utilized as an objective instrument for conducting personalized medicine research incorporating the biopsychosocial perspective.

## 1. Introduction

Personality represents a person's unique pattern of behavior, emotionality, and way of cognition, in addition to specific body features that interact to determine individual's adaptation to the environment [1, 2]. Disease vulnerability or susceptibility and drug response are recognized to be different according to one's personality traits [3–6]. Since provision of personalized medicine has been a major source of concern across cultures, researchers have actively developed medical typologies based on the unique biopsychological pathophysiology, diagnosis, treatment, and prevention mechanism for its clinical application while taking into consideration the influence of cultural and philosophical foundations [7]. The history of temperament related to health (and disease) has a long history in the West and the East. Hippocrates suggested the four humors: blood, yellow bile, black bile, and phlegm as a foundation of physiology. Greek physician Galen followed this tradition and advocated four temperaments or constitutions: sanguine, choleric, phlegmatic, and melancholic, which have been proposed to be akin to stable-extrovert, neurotic-extrovert, stable-introvert, and neurotic-introvert temperaments [7–9]. Furthermore, it has been a major concern in many traditional medicines across the world, such as Ayurveda, Tibetan medicine, and traditional Chinese constitution medicine together with Korean Sasang typology [10]. The Ayurveda from India divides humans into Vata, Pitta, and Kapha types and stems from the temperament of air/wind, fire/water, and water/earth, and provides type-specific meditation, yoga/breathing, aroma therapy, sweating, dietary instruction, and herbal therapy. The Tibetan medicine from the Himalayan region, which is influenced by Tibetan Buddhism and Ayurveda, emphasizes the balance of three Nyipa sum: rLung (vayu), mKhris-pa (pitta), and Bad-Kan (kaph). The Yellow Emperor's Internal Medicine [11] divides the human into five or 25 types with the use of biopsychosocial characteristics based on Yin-Yang and five-phase theory.

The traditional Korean Medicine adopted such perspectives and proposes a systematic medical typology (Table 1) within the quaternary nature of Neo-Confucianism (sadness, anger, gladness, and enjoyment), culminating in the four Sasang types, each with its own characteristic temperament and physical constitution, in addition to behavioral patterns and tendencies, emotionality profile, logical thinking, status of organ system, physiological and pathological features, predisposition to a specific illness, physical characteristics, and response to particular treatments [7, 12]. Individuals are classified into Tae-Yang, So-Yang, Tae-Eum, and So-Eum types, each with their distinctive biopsychological temperaments and type-specific guidelines for safe and effective medical herb and acupuncture use [7, 9, 13]. In other words, Sasang typology embraces the philosophy that an individual's biopsychological characteristics interact to determine his or her response to medical treatment. The So-Yang type is an active and sharp-looking person who is hot-tempered and interested in the outside world, and the So-Eum type is an inactive, prudent, narrow-minded, resolute and nervous [7]. The Tae-Eum type lies in between the So-Yang and So-Eum type in regards to their psychological features and typically has a high body fat mass or body mass index [7, 14, 15]. 

Examination of corresponding Western personality traits in Sasang typology began with the Minnesota Multiphasic Personality Inventory in 1992 and has evolved to the use of various objective tools such as the 16-Personality Factor (16-PF), Myers Briggs Type Indicator (MBTI), State Trait Anxiety Inventory (STAI), Beck Depression Inventory (BDI), Eysenck Personality Questionnaire (EPQ), NEO-Personality Inventory (NEO-PI), and Temperament and Character Inventory (TCI). The theoretical and descriptive similarity of Sasang typology with the Western tradition of personality including Hippocrates and Eysenck has been suggested from these results [9, 14]. More specifically, the two super factors of Eysenck, extraversion and neuroticism, which have been mentioned as critical variables in several personality studies including Costa and McCrae's five-factor model [16], were determined to be important personality constructs of Sasang typology, such that the So-Yang type scored high on the extraversion dimension and low on the neuroticism dimension, while the So-Eum type showed the opposite psychological profile [9]. In the previous studies, the So-Yang type scored high on the NEO-PI extraversion and TCI novelty seeking (NS) subscales and low on the TCI harm avoidance (HA) subscale, and the So-Eum type demonstrated the opposite scores on these assessments [9, 13] (Table 1).

There have been several reports on the influence of personality traits on health [17, 18]. For example, neuroticism or harm avoidance were found to mediate antidepressant response [19, 20], while anxiety alters immunity related to upper respiratory infection [21], natural killer cell activity [22], and functional gastrointestinal disorder [23]. Such association between disease, emotional state, and personality highlights the interplay between biological, psychological, and social factors in determining health status. More recent theoretical framework in explaining personality also relies on such a biopsychosocial model, wherein an individual's personality is defined as an affective state (emotionality) that is associated with expression of feelings and emotions (behavior) that stems from the outcome of individual's appraisal, evaluations, and decisions concerning a particular situation and event (cognition). The measurement of affect includes dimensions of positive and negative affect [24, 25], while the dimension of behavior include such dimensions as approach-action, inhibition-inaction, and flight-fight response [25]. The dimension of cognition includes emotion regulation strategies and broad versus narrow attentional focus [26]. In light of such findings regarding the role of personality in health, it will be important to objectively and systematically examine the personality traits based on Sasang typology. However, little progress was made due to the lack of reliable tools satisfying theoretical and clinical requirements. Hence, the purpose of this study was to develop the Sasang Personality Questionnaire (SPQ) as a novel objective tool to measure the temperaments of each Sasang types based on previous personality studies [9] and examine its psychological construct and validity by comparing the tool with the TCI and NEO-PI. In this course, we may better understand the psychological structure of Sasang typology more systematically and make possible more innovative and originative studies related to the relation between temperament and health. For this purpose, we developed the SPQ and examined it with the TCI and NEO-PI scores of each Sasang type in order to determine the reliability and validity of the SPQ. The SPQ can provide a foundation for the study of individuality on response to medical herbs and acupuncture from a personality perspective, and this study would be able to contribute to scrutinizing the biopsychological traits of Sasang typology and make clinical application and use more efficient. 

## 2. Methods and Materials

### 2.1. Participants and Procedures

Study participants were 245 students from the School of Korean Medicine at Pusan National University and the College of Oriental Medicine at Wonkwang University. This study was approved by the Institutional Review Board (IRB) of the Pusan National University School of Korean Medicine (KCRC IRB 2010-01). All participants gave written consent for the assessments. The mean age of the 245 participants (134 males and 111 females) was 29.01 ± 5.67 (range 21–46).

Since 20 participants did not receive their Sasang type classification based on the Questionnaire for Sasang Constitution Classification II (QSCCII), SPQ was examined with the remaining 225 participants (121 males and 104 females; mean age  28.9 ± 5.7; range 21–46). The mean age of each Sasang type based on the QSCCII was 29.58 ± 6.31 for the So-Yang type (32 males and 31 females), 28.26 ± 5.47 for the Tae-Eum type (40 males and 20 females), and 28.79 ± 5.52 for the So-Eum type (49 males and 53 females).

SPQ item selection using internal consistency, explorative factor analysis using parallel analysis, convergent validity using correlation analysis between SPQ and SPQ subscales, TCI, and NEO-PI were performed (*n* = 225) test-retest reliability of the SPQ and its subscales were analyzed (*n* = 45).

### 2.2. Measures

#### 2.2.1. Questionnaire for Sasang Constitution Classification II (QSCCII)

The QSCCII is a Sasang typology-based inventory, which is composed of 121 forced-choice items including typical diet habits, body shapes, temperaments, and common health problems of each Sasang type. This questionnaire was developed in 1993 and revised in 1996 and has been used as an objective diagnostic tool in many studies examining the biopsychological aspects of Sasang typology [9]. The internal consistency measured with Cronbach's alpha of this inventory is as follows: Tae-yang type is 0.57, So-Yang type is 0.57, Tae-Eum type is 0.59, and So-Eum type is 0.63 [27].

#### 2.2.2. Temperament and Character Inventory (TCI)

The Korean version of the Temperament and Character Inventory-Revised-Short (TCI-RS) [28] is a 140-item self-report questionnaire that asks individuals to rate each item on a 5-point scale (0 = not at all to 4 = very true). TCI is a psychological assessment tool with four temperaments (novelty seeking, harm avoidance, reward dependence, and persistence) and three character dimensions (self-directness, cooperativeness, and self-transcendence) based on Cloninger's biopsychological personality model [29]. The Korean version of the questionnaire was standardized and validated in 2007 and demonstrated validity and reliability. Cronbach's alpha for the novelty seeking, harm avoidance, reward dependence, persistence, self-directness, cooperativeness, and self-transcendence scales were 0.829, 0.857, 0.814, 0.821, 0.865, 0.758 and 0.899, respectively [28].

#### 2.2.3. NEO-Personality Inventory (NEO-PI)

The NEO-PI-R is a 60-item self-report inventory on a 5-point scale (1 = not at all to 5 = very true) based on factor analysis designed to assess a wide spectrum of individual differences, including the universal, stable, and consistent big five structures of neuroticism, extraversion, openness to experience, agreeableness, and conscientiousness [30]. Cronbach's alpha for neuroticism, extraversion, openness to experience, agreeableness, and conscientiousness scale, was reported as 0.850, 0.766, 0.691, 0.644, and 0.720, respectively [31].

### 2.3. Sasang Personality Questionnaire (SPQ) Development

The questionnaire items for the internal consistency analysis were selected from the 42 items collected from the information database of the Sasang typology project (2006-2007) and an online questionnaire development project (2005–2007) of Korea Institute of Oriental Medicine. These questionnaire items were based on Sasang typology theory and descriptions of clinical characteristics of each Sasang type (Table 1). Items that were judged to best represent the biopsychosocial aspects while determined to be the most useful in clinical settings by actual certified specialists based on their clinical experience were finally selected [32].

Each item consists of two opposite words describing a particular personality trait, and participants must respond on a 3-point scale. This response system follows the typical way in which Sasang-type classification is made in clinical practice. For example, the participant can respond as “delicate (= 1),” “average/middle (= 2),” or “tough (= 3)” to the question “Do you have a delicate or tough personality?” The So-Eum type was expected to have a lower score while the So-Yang type evidence higher score on this particular SPQ item based on the consideration of previously reported clinical descriptions of each Sasang type (Table 1).

A review board composed of three traditional medical doctors with more than 5 years of clinical experience and one licensed medical specialist in Korean Sasang typology then selected 15 items that were judged to have a high association with Sasang typology and which may represent typical features of each Sasang type.

The internal consistency of the preliminary 15-item SPQ was examined, after which one item that revealed a low correlation and judged to have low clinical importance was deleted from the final version of the SPQ. That is, the 5th questionnaire item (Q5) was deleted following preliminary item analysis, since the Cronbach's alpha was able to be increased up to 0.817 when deleted. Although the 11th questionnaire item (Q11) was found to increase questionnaire's internal consistency to 0.816 if deleted, this item was decided to be included following panel discussion due to having clinically significant contribution to explaining emotionality of Sasang typology. Therefore, the final 14-item SPQ (Table 2) showed a Cronbach's alpha of 0.817.

### 2.4. Statistical Analysis

#### 2.4.1. Explorative Factor Analysis, Test-Retest Reliability, and Criterion Validity

The 14 items were subjected to explorative factor analysis to examine the possible structure of the SPQ using principal axis extraction and Promax rotation with an eigenvalue over 1.0 as the criteria. We also performed parallel analysis to get the right factor numbers using Monte Carlo PCA for Parallel Analysis which estimates an average distribution of eigenvalues based on a random process that can be compared to the calculated distribution [33, 34]. The meaning of extracted factors was analyzed by reviewing questionnaire items while referring to the factor loadings.

The test-retest data for the reliability examination over a period of one month was analyzed with correlational analysis. The reliability of SPQ and its subscales were determined with Pearson's correlational coefficients. The criterion validity of the SPQ and its subscales were examined by comparing the relationship with the well-established TCI and NEO-PI. We used Spearman's correlational coefficients exceeding the minimum acceptable value of 0.3.

#### 2.4.2. SPQ and Sasang Typology

The SPQ and its subscales (SPQ-B, SPQ-E, and SPQ-C) were subjected to analysis of variance (ANOVA) and profile analysis such as test of parallelism and flatness to examine its effectiveness in representing temperament differences between the Sasang-type groups based on “QSCC II classification”. Demographic differences between Sasang-type groups were tested using ANOVA for continuous variable (age) and Fisher's exact tests for categorical variables (gender).

In addition, we compared the differences of each Sasang-type group on the TCI and NEO-PI to examine if the participants of this study have similar characteristics as in previous studies. ANOVA was conducted to test between group differences in TCI and NEO-PI scores, and Scheffé was used for post hoc analysis, and the profile analysis was performed to test the difference of the TCI and NEO-PI profiles for each Sasang-type group [13].

The data are presented as means and standard deviations or frequency with percentage. All analyses were conducted using PASW Statistics 18.0 for Windows (IBM, Armonk, NY, USA) and *P* value of 0.01 and 0.001 were used for significance.

## 3. Results

### 3.1. Analysis of SPQ and Its Subscales

Promax rotation procedure with SPQ 14 items (Table 2) yielded three interpretable factors. Promax rotation ultimately confirmed three interpretable subscales with cumulative explanatory value of 55.8% of the variance (Table 3).

The first factor, which accounted for 31.5% of the variance, included behavioral components of personality such as “do you consider yourself passive or proactive?” Factor 2, which accounted for 14.9% of the variance, included emotionality component of personality such as “are you relatively patient or impatient?” Factor 3, which accounted for 9.4% of the variance, included personality component related to cognition/decision-making, such as “do you have a delicate or a tough personality?”

The three factors were defined as follows. SPQ-behavior (SPQ-B) measures the behavioral component of personality (passive versus active); SPQ-emotionality (SPQ-E) measures the emotionality component of personality (static versus dynamic); finally, SPQ-cognition (SPQ-C) measures the cognition/decision-making or cognitive component of personality (meticulous versus easy-going). The items comprising each subscale are outlined in Table 2. The Cronbach's alpha of the three subscales from factor analysis was 0.789, 0.685, and 0.711, respectively. Furthermore, the SPQ showed a significant correlation with SPQ-B (*r* = 0.827), SPQ-E (*r* = 0.649), and SPQ-C (*r* = 0.805). SPQ-B showed significant correlation with SPQ-E (*r* = 0.301) and SPQ-C (*r* = 0.541).

The test-retest reliability of the SPQ and its subscales were analyzed with Pearson's correlational analysis. Overall test-retest reliability was found to be 0.837, and reliabilities of the three subscales of SPQ-B, SPQ-E, and SPQ-C were 0.830, 0.748, and 0.798, respectively (Table 4).

The criterion validity of the SPQ was examined with the TCI and NEO-PI. SPQ and its subscales showed significant relations with the TCI and NEO-PI (Table 5).

### 3.2. SPQ and Sasang Typology

The biopsychological characteristics of the participants in this study were analyzed to determine whether the SPQ demonstrates temperament differences between Sasang-type groups and to confirm whether the TCI and NEO-PI scores replicate findings of previous studies [9]. First of all, there were no significant differences between each Sasang type classified with the QSCCII in gender (chi-square = 5,587; *df* = 2;  *P* = 0.061) and age (*F* = 0.831; = 2,213;*P* = 0.437).

**Figure 1:**
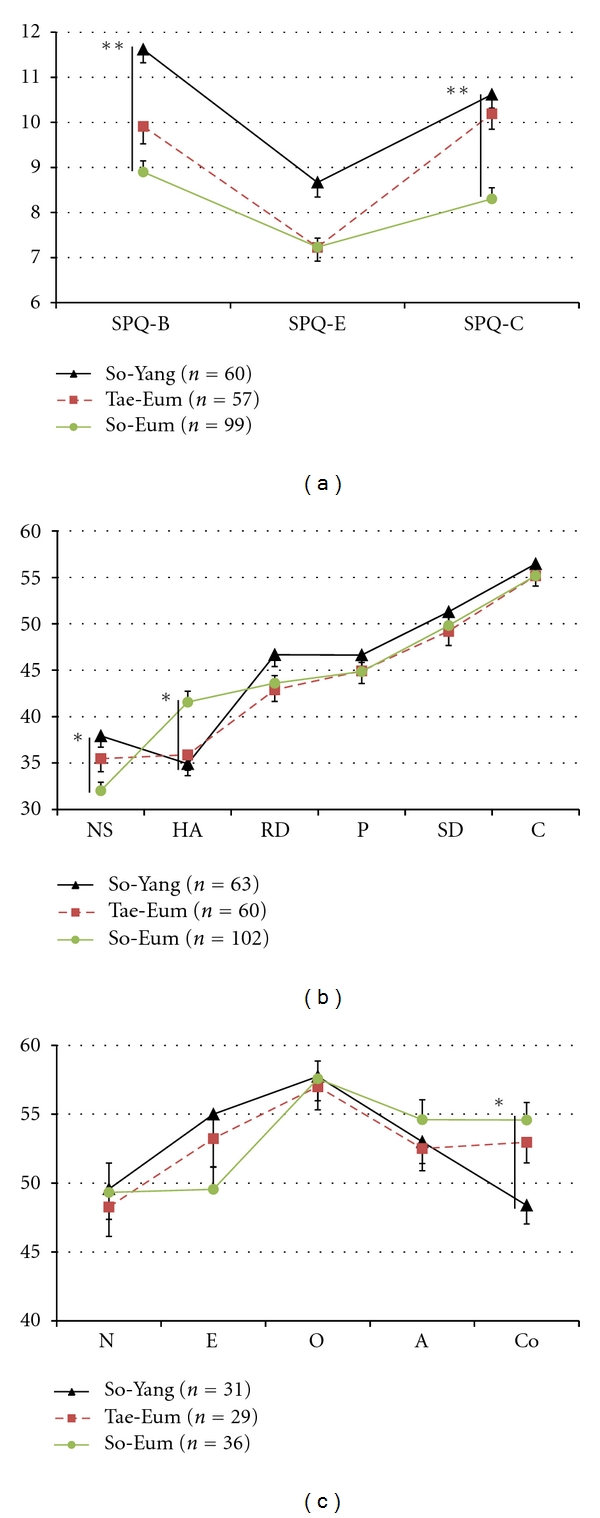
SPQ subscale, TCI and NEO-PI dimension profile of each Sasang type. (a) SPQ subscale score of each Sasang type. The SPQ subscale profile of the So-Yang, Tae-Eum, and So-Eum types was significantly different (flatness with Wilks Lambda test, *df* = 2,*F* = 47.789, and *P* < 0.001; parallelism with Wilks Lambda test, *df* = 4,*F* = 4.920, and *P* = 0.001). The So-Eum type (8.90 ± 2.44, 7.23 ± 1.97, 8.30 ± 2.45) scored significantly (*P* < 0.001) lower on the SPQ-B, SPQ-E, and SPQ-C scales than So-Yang types (11.62 ± 2.27, 8.67 ± 2.5, 10.62 ± 2.32), respectively. (b) TCI score of each Sasang type. The profile of TCI temperament dimension, such as NS, HA, RD, and P was significantly different (flatness with Greenhouse-Geisser test, *df* = 2.542,*F* = 48.867, and *P* < 0.001; parallelism with Greenhouse-Geisser test, *df* = 5.083,*F* = 5.694, and *P* < 0.001). So-Eum type (32.03 ± 9.28, 41.57 ± 11.86) scored significantly lower on NA and higher on HA than So-Yang type (37.92 ± 9.59, and 34.92 ± 10.06) (*P* = 0.001, and *P* < 0.002), respectively. (c) NEO-PI score of each Sasang type. The profile of NEO-PI was not significantly different. So-Eum type (54.58 ± 7.63) scored significantly higher on conscientiousness scale than So-Yang type (48.39 ± 7.49) (*P* = 0.006). *P < 0.01; **P < 0.001. Whisker represents standard errors. SPQ: Sasang Personality Questionnaire, SPQ-B: SPQ-behavior, SPQ-E: SPQ-emotionality, SPQ-C: SPQ-cognition, TCI: temperament and character inventory, NS: novelty seeking, HA: harm avoidance, RD: reward dependence, P: persistence, SD: self directness, C: cooperativeness, ST: self transcendence, NEO-PI: NEO-personality inventory, N: neuroticism, E: extraversion, O: openness to experience, A: agreeableness, Co: conscientiousness.

The SPQ subscales (SPQ-B, SPQ-E, and SPQ-C) of each Sasang type showed significantly different profiles (Figure 1(c)). The profile of the SPQ subfactors, namely, SPQ-B, SPQ-E, and SPQ-C was not flat (Wilks Lambda test: *df* = 2, *F* = 47.789, and *P* < 0.001). As for the parallelism of SPQ subfactor profile with the interaction of Sasang type were significantly different (Wilks Lambda test: *df* = 4,*F* = 4.920, and *P* = 0.001).

Significant differences on the SPQ (*F* = 28.157;*df* = 2,213;*P* < 0.001), SPQ-B (*F* = 21.36;*df* = 2,213;*P* < 0.001), SPQ-E (*F* = 9.019;*df* = 2,213;*P* < 0.001), and SPQ-C (*F* = 20.061;*df* = 2,213;*P* < 0.001) between Sasang types were demonstrated with ANOVA. Post hoc analysis showed that the SPQ of the So-Yang type (30.90 ± 5.23) was significantly higher than that of the Tae-Eum type (27.33 ± 5.88) (*P* = 0.002), and that of the Tae-Eum type was significantly higher than that of the So-Eum type (24.43 ± 4.93) (*P* = 0.005), while SPQ score of the So-Yang type was significantly higher than that of the So-Eum type (*P* < 0.001). Post hoc analysis showed that the SPQ-B of the So-Yang type (11.62 ± 2.27) was significantly higher than that of the Tae-Eum type (9.91 ± 2.93) and the So-Eum type (8.90 ± 2.44) (*P* = 0.002; *P* < 0.001), respectively. Post hoc analysis showed that the SPQ-E of So-Yang type (8.67 ± 2.51) is significantly higher than those of Tae-Eum type (7.23 ± 2.32) and So-Eum type (7.23 ± 1.97) (*P* = 0.003; *P* = 0.001), respectively. Post hoc analysis showed that the SPQ-C of the So-Eum type (8.30 ± 2.45) is significantly lower than those of Tae-Eum type (10.19 ± 2.62) and So-Yang type (10.62 ± 2.33) (*P* < 0.001; *P* < 0.001), respectively.

The TCI temperament (novelty seeking, harm avoidance, reward dependence, and persistence) of each Sasang type exhibited significantly different profiles (Figure 1(a)). The profile of the TCI temperament dimension was not flat (Greenhouse-Geisser test: = 2.542,*F* = 48.867, and *P* < 0.001). As for the parallelism of TCI temperament profile with the interaction of Sasang types, they were significantly different (Greenhouse-Geisser test: *df* = 5.083,*F* = 5.694, and *P* < 0.001). 

The significant differences on the TCI novelty seeking (*F* = 7.315, *df* = 2,222, and *P* < 0.001), TCI harm avoidance (*F* = 7.985,*df* = 2,222, and *P* < 0.001), and TCI reward dependence (*F* = 3.106,*df* = 2,222, and *P* = 0.047) between Sasang types were demonstrated with ANOVA. Post hoc analysis showed that the TCI harm avoidance of the So-Yang type (34.92 ± 10.06) and Tae-Eum type (35.88 ± 12.76) was significantly lower than that of the So-Eum type (41.57 ± 11.86) (*P* = 0.002, and *P* = 0.012, resp.).

The NEO-PI subscales did not show significant profile differences between Sasang types (Figure 1(b)). However, the significant differences on the NEO-PI conscientiousness (*F* = 5.648, *df* = 2,93, and *P* = 0.05) between Sasang types were demonstrated with ANOVA. Post hoc analysis showed that the NEO-PI conscientiousness of the So-Yang type (48.39 ± 7.49) was significantly lower than that of the So-Eum type (54.58 ± 7.63) (*P* = 0.006). The NEO-PI extraversion of the So-Yang type (55.00 ± 10.04) was higher than that of So-Eum type (49.56 ± 9.60), but not statistically significant.

## 4. Discussion

Personalized medicine revolves around the use of safe and effective medical treatment best fit to specific patients, and this theme has been a major concern in both Western orthodox and traditional Eastern medicine. Sasang typology founded in Neo-Confucianism is a temperament-based personalized medicine using acupuncture and medical herbs [10]. In order to provide a more objective and empirical assessment of Sasang-type temperament profile and contribute to broadening diagnostic index for Sasang typology, we developed the Sasang Personality Questionnaire (SPQ), which can measure the psychological traits of Sasang typology, and validated the robust psychological structure of the SPQ in this study.

Reliability and validity examination of the 14-item SPQ demonstrated that the SPQ shows the benefit of multifaceted structural analysis of Sasang typology across the domains of behavior, emotionality, and cognition with acceptable reliability and stability [35]. Furthermore, the biopsychological features of each Sasang type when compared with the TCI and NEO-PI replicated previous clinical studies [9, 13, 36–38], and such results further illustrate the usefulness of the SPQ in clinical use.

It was found that the SPQ measures three facets of personality associated with Sasang typology, namely, behavior, emotionality, and cognition. More specifically, the So-Eum type scored relatively lower on the SPQ such that its personality can be characterized as passive (behavior), static (emotionality), and meticulous (cognition). In contrast, the personality profile of the So-Yang type can be characterized as active (behavior), dynamic (emotionality), and easy-going (cognition) as indicated by a higher score on the SPQ. From the perspective of traditional Korean medicine, such characteristics of the SPQ subscales reflect the basic theoretical foundation of Eastern philosophy that the smaller change in behavior, emotionality, and cognitive characteristics of the SPQ mirror the Yin (So-Eum type), while greater change mirrors the Yang (So-Yang type).

If we sum up these results, SPQ is a reliable and objective psychological measurement of the So-Eum : Tae-Eum : So-Yang axis as suggested in previous studies [14] and has theoretical connections with the Western psychological discipline [9], which is useful for further comparative studies. The lower extreme of the Sasang personality axis was again found to be the So-Eum type, which exhibits low novelty seeking and high harm avoidance on the TCI, and low extraversion on the NEO-PI, while at the other extreme lies the So-Yang type which demonstrates opposite psychological features. The Tae-Eum type is located in the middle and has a higher body mass index compared to both the So-Yang and So-Eum types. 

The psychological structure of the SPQ subscales was compared with existing validated psychological instruments using correlational analysis. The results indicated that the SPQ and its subscales are generally in the same direction as the TCI. However, it was found that the SPQ-emotionality subscale demonstrated a positive correlation with NEO-PI neuroticism, but the SPQ-behavior and SPQ-cognition subscales showed negative correlations (Table 5). The NEO-PI neuroticism is a sum of heterogeneous traits [39] and operationally defined as irritability, anger, sadness, impulsiveness, vulnerability, hostility, and worry [40–42]. Although features of SPQ-emotionality items including being impatient, illogical, and greater emotional change appear related to the construct of neuroticism, the SPQ-behavior and SPQ-cognition items representing proactiveness, fluency and directness in expression of personal opinion, energetic nature, and tough personality seem contradictory to the traditional characteristics of neuroticism (Table 2).

This disparity could be the reason why current and previous studies [38] failed to show significant differences in NEO-PI neuroticism between Sasang types, even though neuroticism has been suggested as the major axis of Sasang typology with indirect evidences from studies using the STAI, BDI, and other assessment tools [9]. Further studies are needed to determine medical implications of such differences between NEO-PI neuroticism and emotionality, behavior, and cognition on the SPQ that was founded on Eastern medicine.

As for the behavioral, emotional, and cognitive facets of the Sasang personality in this study, the manner in which an individual will evaluate a particular situation or environment will be determined by the individual's emotional state, which will in turn be expressed in various corresponding behavioral forms judged to be suitable to the situation or event. For example, the positive affect-negative affect dimension has emerged as an important dimension of an individual's emotional experience, such that self-estimate of such affect results in expressions of mood that correspond to measures of personality and emotionality, in addition to making possible predictions of cognitive performance [43]. The associations between extraversion and positive affect and between neuroticism and negative affect are well documented [44]. In one study, it was found that the personality construct of extraversion and neuroticism contributed to negative and positive affects indirectly through emotional reappraisal, suggesting that depending on the type of personality dimension an individual holds, different emotion regulation strategies or cognitive appraisal styles might exist [45].

Since there are insufficient studies examining the biopsychological basis and its medical value in different medical typologies, more scrupulous and practical studies are needed. Current studies with conventional Western medicine have reported that specific temperaments make a person predisposed to certain disease and affect its prognosis. For example, although much disagreement still exists, the link between cancer and type C personality was suggested wherein a personality that suppresses emotions and shows difficulty in coping with stress [46], and a possible association between coronary heart disease and type D personality, where one demonstrates negative affectivity and social inhibition [47, 48] have been proposed.

The SPQ and its subscales (SPQ-behavior, SPQ-emotionality, and SPQ-cognition) showed three facets of Sasang personality and provide further rationale for how one's personality may affect health in clinical health psychology studies [18, 49]. The personality trait of extraversion, for instance, can be characterized by positive affect, approach behavior, and broad focus, while the trait of neuroticism can be marked by negative affect, inhibition of behavior, and narrow focus. It will be important to examine the association of such a tripartite model with disease vulnerability, progress, and outcome across the Sasang types.

The relationship between stress and anxiety (neuroticism) has gained much interest in recent days. Predisposition of the HPA axis related to the stress response, the functioning of the stress-related response by the interleukin and natural killer cell have also been proposed as potential mediators in the stress-response cycle [50]. The hostility score on the Minnesota Multiphasic Personality Inventory (MMPI), negative affectivity of the Positive and Negative Affect Scale (PANAS), neuroticism of the fives factor model, and optimism determines the stress response which ultimately affects health-related pathophysiology [51], and neuroticism and extraversion have been reported to have influence on morbidity and mortality [4, 18, 49]. For example, this can be related to studies on stress response in that threatening or frustrating stimuli may activate the fight-flight-freezing system (FFFS), which produces active avoidance (anxiety and flight) or attempted elimination (anger and attack). Even stimuli or situations that contain potential threat can activate the behavioral inhibition system (BIS), which may result in vigilance, rumination, and passive avoidance as well as anxiety [52]. The constructs of “agentic extraversion,” characterized by assertiveness, dominance, and ambition, have been distinguished from “affiliative extraversion,” characterized by sociability and affiliative bonding [53] are similar in content to the items of our SPQ-cognition subscale.

This study has several limitations that may affect generalizability. First, this study should be repeated with a larger and more balanced sample size. Although the TCI and NEO-PI scores of each Sasang-type groups in this study were not significantly different compared to previous studies [13, 38], the prevalence (So-Yang : Tae-Eum : So-Eum) of Sasang-type groups in this study was 2 : 2 : 3, not the proposed 2 : 5 : 3 described in the previous studies, which may have resulted in the possibility of selection bias from the difference in Sasang-type group sample size ratio [7, 13]. There also should be studies considering age and gender differences [14] in Sasang typology if the SPQ is to be used as a generalized measure of the Sasang personality construct.

Second, SPQ-behavior, SPQ-emotionality, and SPQ-cognition were extracted as the subscales of SPQ, and these should be validated with diverse psychological instruments alongside with their stability. In particular, as the SPQ-emotionality correlated with NEO-PI neuroticism while, SPQ, SPQ-behavior, and SPQ-cognition negatively correlated with NEO-PI neuroticism, other assessments much are examined to see whether such patterns continue to hold. The reason for distinct differences in emotionality or neuroticism dimension between Western psychology and Sasang typology should be analyzed to determine if such differences result from cultural or philosophical disparities, clinical experience, personality construct, or other factors [54].

Third, the possibility of response bias, or the tendency of participants to provide socially acceptable responses, cannot be ruled out, especially as they must self-assess using the SPQ. However, this may have been minimized due to the forced-choice nature of the SPQ and the fact that participants were unaware of its scoring method. In addition, the relatively high test-retest reliability score of the SPQ and the replication of theory-driven association with the NEO-PI and TCI suggest that the likelihood of response bias may have been reduced in the present study.

 Last but not least, the clinical usefulness of SPQ should be substantiated using clinical samples. Although the psychological and biometric profiles of each Sasang type in this study using the QSCCII are a replication of previous clinical studies [13, 38], there still lies a possibility of age, gender, and socioeconomical characteristics that together may affect Sasang-type classifying method. A future study testing whether the SPQ can predict individual's response to acupuncture and medical herbs with various age groups in clinical situations should be conducted.

In conclusion, the Sasang Personality Questionnaire which can objectively measure the psychological personality basis of Sasang typology was developed and validated with Western psychometric instruments in this study. With further clinical investigation, the SPQ may serve as a solid foundation for personalized medicine with medical herbs and acupuncture by providing a biopsychosocial typology perspective.

## Figures and Tables

**Table 1:** Characteristics of the Sasang typology (modified from the previous studies of Chae et al. [7, 9] and Park et al. [13]).

Type (prevalence)	Tae-Yang (*太陽*) (<0.1%)	So-Yang (*少陽*) (20%)	Tae-Eum (*太陰*) (50%)	So-Eum (*少陰*) (30%)
Natural temperament	Sorrow (*哀*) by benevolence (*仁*)	Anger (*怒*) by righteousness (*義*)	Gladness (*喜*) by courtesy (*禮*)	Enjoyment (*樂*) by wisdom (*智*)

Organ system	Developed lung and undeveloped liver	Developed spleen and undeveloped kidney	Developed liver and undeveloped lung	Developed kidney and undeveloped spleen
	Developed consumption and catabolism	Developed intake and digestion	Developed accumulation and anabolism	Developed waste discharge

Representative features	Masculine, forward moved, and originative	Active, external-oriented, talented for business, short, and small	Feminine, stay retracted, conservative, tall, and big	Still, internal-oriented, self-directed, short, and small

Character	Creative, positive, progressive, charismatic, heroic, and rash mind	Unstable, easily get bored, sacrificing, righteous, easily acceptable, hot-tempered, and anxious mind	Gentle, commercial, endurable, humorous, look foolish, coward, and fearful mind	Neat, mild, negative, intelligent, organized, selfish, jealous, persistent, and nervous mind
		High extraversion and low neuroticism (NEO-PI)high novelty seeking and low harm avoidance (TCI)		Low extraversion and high neuroticism, low novelty seeking, and high harm avoidance

Body shape	Developed nape of the neck and slender waist	Developed chest and small hips	Thick waist, weak nape of the neck	Developed hip and weak chest
		Low BMI and waist-hip ratio, low width-height ratio of face, and smaller neck circumference	High BMI and waist-hip ratio. High width-height ratio of face. Bigger neck circumference	Same as So-Yang type, but more smaller and slimmer

Sign for healthy and unhealthy condition	Good urination,	Good bowel movement	Good perspiration	Good digestion
	bubbles in mouth, and emesis	Constipation	No perspiration	Indigestion
			Prone to diabetes and high-insulin resistance. More perspiration than others	Frequent indigestion and upper respiratory infection

Type-specific useful medical herbs	Chaenomelis Fructus, Acanthopanacis Cortex, and Phragmitis Rhizoma	Rehmanniae Radix, Corni Fructus, Hoelen, Alismatis Rhizoma, Osterici Radix, and Angelicae Pubescentis Radix	Ephedrae Herba, Liriopis Tuber, Schisandrae Fructus, Dioscoreae Rhizoma, Platycodi Radix, Coicis Semen, and Puerariae Radix	Ginseng Radix, Atractylodis Rhizoma Alba, Glycyrrhizae Radix, Cinnamomi Cortex, Citri Pericarpium, Zingiberis, and Rhizoma Crudus

Type-specific acupuncture use	Diagnosis with HT8 Treatment with LR3(+)/LU9(−)	Diagnosis with HT3 Treatment with KI3(+)/SP3(−)	Diagnosis with HT4 Treatment with LU9(+)/LR3(−)	Diagnosis with HT7 Treatment with SP3(+)/LI4(−)

**Table 2:** Extracted and rotated factor-loading matrix of SPQ subscales and questionnaire items.

	Questionnaire items	Factor loading		
		**1**	**2**	**3**
SPQ-behavior (passive/active)	Q3: do you consider yourself passive or proactive?	**0.761**	−0.076	0.177
	Q6: is your personality relatively introverted or extroverted?	**0.721**	0.234	0.289
	Q7: do you consider yourself relatively lethargic or energetic?	**0.693**	0.179	0.246
	Q2: are you relatively slow or quick?	**0.691**	−0.059	0.013
	Q13: do you tend to not express your opinions or express well?	**0.582**	0.36	0.257

SPQ-emotionality (static/dynamic)	Q10: are you relatively patient or impatient?	−0.028	**0.754**	0.154
	Q14: do you tend to be logical or do you sometimes get excited?	0.082	**0.722**	0.049
	Q11: do you tend to experience little emotional change or big emotional change?	0.042	**0.714**	−0.269
	Q12: do you consider yourself as someone who expresses inner thoughts and feelings a little or a lot?	0.37	**0.559**	0.094

SPQ-cognition (meticulous/easygoing)	Q1: do you have a delicate or tough personality?	0.172	−0.007	**0.813**
	Q9: do you consider yourself feminine or masculine?	0.26	−0.004	**0.721**
	Q15: do you tend to act meticulously or hastily?	−0.122	0.488	**0.579**
	Q8: in general, do you make decisions with difficulty or with ease?	0.346	−0.141	**0.524**
	Q4: do you tend to be relatively indirect or direct when expressing yourself?	0.36	0.327	**0.51**

**Table 3:** Extraction of SPQ subfactors with explorative factor analysis using Promax rotation.

Factor	Extraction sumsof squared loadings			Rotation sums of squared loadings		
	Eigenvalue	percent of variance	Cumulative percent	Eigenvalue	percent of variance	Cumulative percent
1	4.410	31.501	31.501	2.904	20.744	20.744
2	2.086	14.904	46.405	2.503	17.882	38.625
3	1.311	9.362	55.767	2.400	17.141	55.767

**Table 4:** Test-retest reliability for the SPQ and its subscales.

	Score of the test	Score of the retest	Pearson's correlation coefficient
SPQ	27.31 ± 5.84	26.82 ± 5.71	.837**
SPQ-Behavior	10.33 ± 2.71	10.11 ± 2.79	.830**
SPQ-Emotionality	7.53 ± 2.27	7.47 ± 2.08	.748**
SPQ-Cognition	9.44 ± 2.75	9.24 ± 2.30	.798**

**P < 0.001.

**Table 5:** Correlation between SPQ, subscales of SPQ, TCI, and NEO-PI.

	SPQ (*n* = 232)			TCI (*n* = 232)							NEO-PI (*n* = 97)				
	SPQ-B	SPQ-E	SPQ-C	NS	HA	RD	P	SD	C	ST	N	E	O	A	Co
SPQ	0.827**	0.649**	0.805**	0.462**	−0.390**	0.320**	0.225**	0.148	0.026	0.135	−0.168	0.629**	0.182	−0.171	−0.183
SPQ-B		0.301**	0.541**	0.314**	−0.513**	0.334**	0.403**	0.358**	0.114	0.190*	−0.320*	0.683**	0.192	−0.081	0.108
SPQ-E			0.265**	0.364**	0.137	0.408**	−0.099	−0.226**	−0.043	0.061	0.339**	0.288*	0.094	−0.036	−0.326*
SPQ-C				0.387**	−0.452**	0.012	0.167	0.153	−0.023	0.049	−0.329*	0.452**	0.129	−0.263*	−0.236

*P < 0.01; **P < 0.001. Bold represents correlation coefficient over 0.3.

SPQ: Sasang Personality Questionnaire, SPQ-B: SPQ-behavior, SPQ-E: SPQ-emotionality, SPQ-C: SPQ-cognition, TCI: temperament and character inventory, NS: novelty seeking, HA: harm avoidance, RD: reward dependence, P: persistence, SD: self directness, C: cooperativeness, ST: self transcendence, NEO-PI: NEO-personality inventory, N: neuroticism, E: extraversion, O: openness to experience, A: agreeableness, Co: conscientiousness.
